# Sensory evaluation of selected formulated milk barberry drinks using the fuzzy approach

**DOI:** 10.1002/fsn3.454

**Published:** 2017-01-13

**Authors:** Zahra Tahsiri, Mehrdad Niakousari, Sara Khoshnoudi‐Nia, Seyed Mohamad H. Hosseini

**Affiliations:** ^1^ Department of Food Science and TechnologySchool of AgricultureShiraz UniversityShirazIran

**Keywords:** functional drinks, fuzzy decision making, milk‐barberry drinks, pectin, sensory evaluation

## Abstract

Amid rigid competition in marketing to accomplish customers' needs, the cost of disappointment is too high. In an effort to escape market disappointment, one of the options to be considered is probing for customer satisfaction through sensory evaluation. This study aims to rank the six selected milk‐barberry drink formulae out of 24 (code numbers S3, S4, S15, S16, S17 and S18) each having different milk:barberry:pectin amount (7: 3: 0.2; 6: 4: 0.2; 7: 3: 0.4, 6: 4: 0.4, 5: 5: 0.4 and 6: 4: 0.4), respectively, and to determine the best of quality attribute through sensory evaluation, using the fuzzy decision‐making model. The selection was based on pH, total solid content, and degree of serum separation and rheological properties of the drinks. The results showed that the S4 had the highest acceptability, rated under the “very good” category, whereas the lowest acceptability was reported for the S3 which was classified under the “satisfactory” category. In summary, the ranking of the milk‐barberry drinks was S4 >  S17 >  S16 >  S15 >  S18 >  S3. Furthermore, quality attributes were ranked as taste > mouth feel > aroma > color. Results suggest that the fuzzy approach could be appropriately used to evaluate this type of sensory data.

## 1 Introduction

Antioxidants principally function to reduce oxidizing damages, as these damages contribute to the cause of cardiovascular disease, Alzheimer's, cancer, cataract and diabetes (Rodríguez‐Roque, Rojas‐Graü, Elez‐Martínez, & Martín‐Belloso, 2013; Slattery et al., 2000). Fruits and vegetables contain different bioactive compounds such as vitamins A, C, and E. Phenolic compounds are also found in fruits with antioxidant activities, and have shown to be good contributors to the total antioxidant capacity of the food that contain them (Chaovanalikit & Wrolstad, 2004). Barberry is rich in anthocyanin and vitamin C. Anthocyanins are polyphenol compounds which belong to the group of water soluble pigments that can be used for food coloring (Harborne, 2013). Recently, considerable attention has been directed to nutraceutical foods which have led breeders to initiate the selection of plants with antioxidant capacities being higher than the normal. In this context, then, the barberry is a suitable plant for relevant investigation. Formulating milk by barberry juice, pectin, sugar, and producing acidified milk drinks not only increases the nutritional and pharmaceutical properties of milk but also has the potential to increase sales and promote the appeal of milk. Furthermore, it can be applied in different products such as in confectionaries and ice cream products or be used as a natural color as an alternative to artificial food coloring. A large range of drinks from those are prepared from fermented milk with stabilizers added to those prepared by direct acidification with fruit juices and/or acids which render acidified milk drinks. He et al. investigated the effect of pH adjustment and thermal treatment on the antioxidant capacity of fruit juice beverages and results revealed that pasteurization (63°C/30 min) and pH adjustment (pH 3.7 or 6.8) had either non‐ significant or slight effects on fruit juice milk beverage's antioxidant capacity (He, Yuan, Zeng, Tao, & Chen, 2015). The pH of acidified milk drinks (AMDs) range from 3.6 to 4.2 and this could be accompanied by sedimentation problems and subsequent macroscopic whey separation in this pH range. Relevant to this context, Janhøj et al. produced drinking yoghurt made from fruit concentrate and reconstituted milk powder, and then the drinks were stabilized with pectin and/or carboxymethyl cellulose (CMC) (0–0.5%) (Janhøj, Frøst, & Ipsen, 2008). Caseins are assumed to be micelles at the neutral pH in milk. Caseins remain in suspension form due to the hairy layer of k‐casein which provides steric and electrostatic repulsive interactions between casein micelles. These interactions cause caseins to stay in their suspended state (De Kruif, 1998; Holt, 1992; Schmitt et al., 2000). However, this mechanism of stabilization of casein micelles fails to be maintained in the pH value around 4, due to the collapse of the extended conformation of k‐casein. The tendency to increase entropy of k‐casein chains causes the repulsive interaction between k‐casein chains because the k‐casein chains of neighboring micelles tend to overlap and would result in the loss of entropy of the chains. This phenomenon is called steric stabilization (Tuinier, Rolin, & De Kruif, 2002). In dilute acidified milk systems, pectin was added to AMDs with less than 1% (w/w) nonfat milk solids as the stabilizer. It was shown that pectin is adsorbed onto the casein micelles because of electrostatic interaction (Glahn & Rolin, 1994). Appearance is the foremost criterion that influences the acceptance or rejection of food by consumers; therefore, stabilizers are widely used to stabilize these drinks to prevent the flocculation of milk protein. This assists in the attainment of optimal mouth feel, thereby enhancing the favorable features of products (De Kruif & Tuinier, 2001; Glahn & Rolin, 1994). Sensory qualities of foods can be evaluated based on estimations of the total impression the food makes on the mind of the person consuming the food (Giusti, Bignetti, & Cannella, 2008; Reinoso, Mittal, & Lim, 2008). The sensory evaluation of food is often regarded as being characterized by inaccuracy, mistakenness and uncertain repeatability. Nonetheless, sensory data, viz. appearance, taste, mouth feel, aroma, and color are normally analyzed statistically, and yet it is not possible to find out precisely by such analyses the strength and weakness of specific sensory attributes which are mainly involved in determining the acceptance or rejection of the drinks. This shows the importance of such decision‐making as a tool for ranking the quality of products evaluated by the panelists. It is also a method which aids in comparing new products with similar products already in markets. According to earlier reports by researchers, the Fuzzy logic is a useful tool that can be employed when conducting analyses on sensory data of many food products like drinks (Lazim & Suriani, 2009).

The present article was undertaken to elucidate the acceptable level of ingredients which can be incorporated into the production of milk barberry juice drink with the greatest stability. The objective is achieved by analyzing evaluations on physicochemical data. This article also aims to investigate the quality of produced AMD samples through sensory evaluation by ranking the AMD samples with respect to their quality attributes, using the fuzzy logic. Attempts were also made to find out the strength and weakness of each sample.

## 2 Materials and Methods

All chemicals were of analytical grade and were purchased either from Merck (Darmstad, Germany) or Sigma–Aldrich (St. Louis, MO, USA). In order to prepare barberry juice (20°Bx), seedless barberry (*Berberis vulgaris*) was purchased from a local market in Shiraz, Iran. Low fat milk was purchased from Pegah Fars Dairy Company (Shiraz, Iran). To formulate the AMDs, GENU® Pectin type YM‐150‐L (with an esterification degree of 72%) was purchased from CP Kelco (Lille Skensved, Denmark).

### 2.1 Preparation of barberry juice concentrate

The stalks of seedless barberry (*Berberis vulgaris*) were detached from the fruits which were then stored in cold storage at −18°C. Before extracting the juice, the barberries were washed. The juice was mixed with distilled water (5: 1). This mixture was then exposed to ultrasound Bandelin (DT255 H, BANDELIN electronic GmbH and Co, Berlin, Germany) at 35°C. The barberry juice was subsequently transferred to a Kenwood blender (Blend‐X Classic BLP607WH, Havant, England) and was mixed for 5 min at 1,000 rpm. Finally, the mixture was concentrated in a rotary evaporator at 30°C (Model R‐3, BUCHI Co, Flawil, Switzerland) to reach 20°Bx.

### 2.2 Chemical analysis of barberry concentrate

#### 2.2.1 Antioxidant activity

In this study, the total antioxidant activity, anthocyanins content, and total phenolic compound of the barberry juice concentrate (BJC) was determined.

Free radical scavenging activity (RSA) of the BJC was determined according to the method of Cam, Hisil, and Durmaz (2009). A volume of 0.1 ml of samples was mixed with 0.9 ml of 100 mmol/L Tris‐HCl buffer to which 1 mM of DPPH was added and vortexed. The reaction mixture was left in the dark for 30 min, after which the absorbance of the resulting solution of BJC was recorded by a Reyleigh spectrophotometer (Model VIS‐7220G/UV9200, Beijing Beifen‐Ruili Analytical instrument (Group) Co, Beijing, China) at 517 nm. The control sample was prepared in a similar way by adding 0.1 ml of water instead of juice sample. The antioxidant activity was expressed in the percentage of inhibition of the DPPH radical, and was determined by the following equation:

(1) $$\text{RSA}\%=[\left(A_{\mathrm{control}}-A_{\mathrm{sample}}\right)/A_{\mathrm{control}}]\times 100$$

where *A*_sample_ is the barberry concentrate absorbance at 517 nm and *A*_control_ is the control sample absorbance at 517 nm (Çam, Hışıl, & Durmaz, 2009).

#### 2.2.2 Total anthocyanin measurement

The total anthocyanin content of BJC was determined by the pH differential method based on structural transformations of anthocyanins as a function of pH‐generating color solutions. The flaviliccation exhibits red color and is the most prominent form of its type in pH 1.0, while carbinol is colorless and is most abundant at pH 4.5. In this method, two buffer systems are used: the potassium chloride buffer pH 1.0 (0.025 mol/L) and the sodium acetate buffer pH 4.5 (0.4 mol/L) (Cam et al., 2009). Briefly, 0.4 ml of the BJC sample was mixed with 3.6 ml of the corresponding buffer and was read against water, as the blank, at 510 nm (A510) and 700 nm (A700). Absorbance was determined by the following equation:

(2) $$A={\left(A510-A700\right)}_{\mathrm{pH}1.0}-{\left(A510-A700\right)}_{\mathrm{pH}4.5}$$

Total anthocyanins content (TAC) of the sample (mg cyanidin‐3‐glucoside/100 ml of BJC) was calculated by the following formula:

(3) TAC=(A×MW×DF×100)/MA

where A is the absorbance, MW is the molecular weight (449.2), DF is the dilution factor (10), and MA is the molar absorptivity of cyanidin‐3‐glucoside (26.900).

#### 2.2.3 Total phenolic content measurement

Total phenolic content in extracts was determined by Folin–Ciocalteu colorimetric method according to Vinson et al. (1995); 0.5 ml of the concentrated juice sample was mixed with 2.5 ml folin–ciocaltieu as the chemical reagent (0.2 N) and the resultant solution was poured into each tube. The solutions inside the tubes were mixed by a shaker for 30 s, and then 2 ml of sodium carbonate 7.5% was added after 3 min of rest. The solution was further mixed for 30 s at ambient temperature for 1 hr. Absorption of each sample was read at 765 nm by a spectrophotometer. The amount of phenol components can be calculated by the calibration of gallic acid as the standard (Vinson, Dabbagh, Serry, & Jang, 1995).

#### 2.2.4 Vitamin C measurement

The vitamin C was quantified using the Knauer 1000 high‐performance liquid chromatography (HPLC) (Knauer Corp., Berlin, Germany), which was equipped with Nucleodur, C18 pyramid (250 × 4.6 mm, 5 μm, Germany) fitted with the same guard column. A gradient of mobile phase created of methanol (solvent A) and 5 mmol/l KH_2_PO_4_, pH 2.65 (solvent B) was used according to the following program: linear increment starting with 5%–22% A in 6 min and the return to the initial conditions within the next 9 min with the flow rate of 0.8 ml/min. The eluate was detected, using a Knauer 2600 photodiode array detector set at 245 nm (Gliszczyńska‐Świgło et al., 2006). The injection volume was 20 μL.

### 2.3 Preparation of milk‐barberry drink

AMDs were prepared by mixing the milk‐barberry (Milk: Barberry) in ratios of 9: 1, 8: 2, 7: 3, 6: 4, 5: 5, 6: 4 with high methoxyl pectin solutions at concentrations of 0.2%, 0.3%, 0.4% and 0.5%. Accordingly, twenty‐four formulae were tested in a 6 × 4 design in two levels of homogenized and nonhomogenized solutions (with two variables: Milk: Barberry ratio and pectin concentration). For this purpose, 0.2%, 0.3%, 0.4% and 0.5% pectin solutions were prepared from high methoxyl pectin (HMP) at 75°C and were stored overnight at 4°C. The proper amount of milk was tempered in a 40°C water bath added to the pectin solutions and was mixed by rotating at 500 rpm for 5 min on the magnetic stirrer. Then, the specified amount of BJC and 8% sugar were added. All samples were stirred and subsequently homogenized at 10,200 rpm for 2.5 min (IKA T 25 digital ULTRA‐TURRAX) and were then pasteurized at 72°C for 15 s and stored in sealed plastic centrifuge tubes at 4°C for 10 days. Table 1 depicts all the formulations related to the production of milk‐barberry concentration beverage.

**Table 1:** Specification of different formulation ^(^ a ^)^ used to determine the best formula for produced milk‐barberry drinks

Formulation code	Ratio milk to barberry juice concentrate	Barberry juice concentrate, g	Milk, g	Pectin, g
S1	9:1	9.18	82.62	0.20
S2	2:8	18.36	73.44	0.20
S3	7:3	27.54	64.26	0.20
S4	6:4	36.72	55.08	0.20
S5	5:5	45.90	45.90	0.20
S6	4:6	55.81	36.72	0.20
S7	9:1	9.17	82.53	0.30
S8	2:8	18.34	73.36	0.30
S9	7:3	27.51	64.19	0.30
S10	6:4	36.68	55.02	0.30
S11	5:5	45.85	45.85	0.30
S12	4:6	55.02	36.68	0.30
S13	9:1	9.16	82.44	0.40
S14	2:8	18.32	73.28	0.40
S15	7:3	27.48	64.12	0.40
S16	6:4	36.64	54.96	0.40
S17	5:5	45.80	45.80	0.40
S18	4:6	54.96	36.64	0.40
S19	9:1	9.16	82.44	0.50
S20	2:8	18.32	73.28	0.50
S21	7:3	27.48	64.12	0.50
S22	6:4	36.64	54.96	0.50
S23	5:5	45.80	45.80	0.50
S24	4:6	54.96	36.64	0.50

a It is necessary to mention that in all formulations, fixed amount of sugar was considered (i.e., equal to 8 g).

### 2.4 Physicochemical properties of milk‐barberry drink

#### 2.4.1 PH measurements

The pH of samples were monitored immediately after production (pH/mV, model PT370, Keison, UK).

#### 2.4.2 Total solid measurements

Total amount of dry solid is determined by the equation TS=100‐w.b.(%) (Ahmed, Ramaswamy, & Khan, 2005).

#### 2.4.3 Stability measurement

To monitor stability, 50 ml plastic centrifuge tubes were filled with samples and were stored at 4°C. After 10 days of storage, the weight of the layer of whey, if present, was measured and the percentage (w/w) of whey separation was calculated. If the percentage of whey separation is more than 10%, the sample is considered unstable.

#### 2.4.4 Viscosity measurement

Viscosity of samples was evaluated, using the Brookfield Viscometer (RVDV‐ II Pro, Brookfield Engineering, Massachusetts, USA), equipped with spindle HA3 running at 10, 30, 60 and 100 rpm. Measurements were taken at 4°C.

#### 2.4.5 Color measurement

The color of samples was measured, using digital imaging and the Photoshop software (Adobe system Inc., san Joes, California, USA) (Afshari‐Jouybari & Farahnaky, 2011).

### 2.5 Statistical analysis

All data (except sensory data that analyzed by fuzzy logic) were statistically analyzed, using the analysis of variance (ANOVA) procedure of the Minitab 16 (, State College, PA, USA). The analysis was carried out in three replications. Tukey's multiple range tests were applied to determine significance of differences between mean values (*p* < .05).

### 2.6 Sensory analysis

Initially, six milk barberry drink samples were evaluated based on the desirability values of pH, and sensory evaluation was applied via Serum Separation. A panel of twenty healthy panelists (eleven females and nine males) was selected from among the staff members and students of the Food Science and Technology Department, Shiraz University, in order to assess successive milk‐barberry drinks. Initially, the panel was familiarized with the various terminology employed to describe the sensory evaluation (color, taste, aroma, and mouth feel), and the score sheet and method of scoring. The panelists were asked to give check marks (⋅) to the appropriate respective fuzzy scale factor for each sample after evaluating the milk‐barberry comprehensively. The judgments were to be made quickly but not hurriedly. The panelists were asked to rinse their mouth with water after tasting each milk‐barberry sample. The samples were rated as “Poor,” “Fair,” “Good,” “Very Good” or “Excellent. The sets of observations were analyzed, using Fuzzy analysis of sensory scores (Jaya & Das, 2003; Meena, Gupta, Khetra, & Raghu, 2015; Routray & Mishra, 2012).

#### 2.6.1 Fuzzy analysis of sensory data

**Figure 1:**
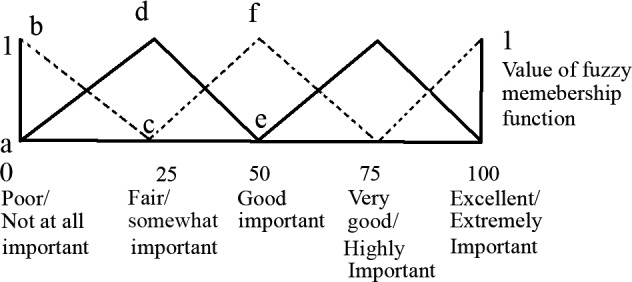
Triangular membership function distribution pattern of 5‐point scale (Jaya & Das, 2003; Sinija & Mishra, 2011)

This method utilizes linguistic data obtained by sensory evaluation. Ranking of the milk‐barberry samples was performed using the triangular fuzzy membership distribution function as described by Sinija and Mishra (2011). Sensory scores of the milk‐barberry samples were obtained, using the fuzzy scores provided by the panelists, which were converted to triplets and used for the estimation of similarity values employed in the ranking of samples. The major steps involved in the fuzzy modeling of sensory evaluation were: (1) calculation of overall sensory scores triplets of the milk‐barberry; (2) computation of membership function on standard fuzzy scale; (3) estimation of overall membership function on standard fuzzy scale; (4) estimation of similarity values and ranking of milk‐barberry samples. A program in Matlab 2015a (The Math Works) was developed for the calculation of all the above‐mentioned steps. Triangular membership function distribution pattern of 5‐point sensory scales were delineated by a set of three numbers, known as the “triplet”. The distribution pattern of 5‐point sensory scales are composed of “Poor, (0, 0, 25)”, “Fair, (25, 25, 25),” “Good, (50, 25, 25)” “Very Good (75, 25, 25)” and “Excellent (100, 25, 0)”. The first number of the three numbers shown in the parentheses denotes the coordinate of the abscissa where the value of the membership function is 1 (Figure 1), and the second and third numbers of the triplet designate the distance to the left and right, respectively, of the first number, where the membership function is zero (Sinija & Mishra, 2011).

#### 2.6.2 Triplets for sensory scores of milk‐barberry drinks and overall quality

The triplet (three number set) for sensory scores of each quality items of every sample can be calculated by the following equation:

(4) $$S_{r}C=\frac{n_{1}\left(0\;0\;25\right)+n_{2}\left(25\;25\;25\right)+n_{3}\left(50\;25\;25\right)+n_{4}\left(75\;025\;25\right)+n_{5}\left(100\;25\;0\right)}{n_{1}+n_{2}+n_{3}+n_{4}+n_{5}},$$

where (n1 +  n2 +  n3 +  n4 +  n5) resembles the total number of panelists, C is for the color item, the subscript “r” is for sample number while n1, n2, n3, n4 and n5 are the number of panelists who give “Poor”, “Fair”, “Good”, “Very Good” or “Excellent” scores to each quality attribute of each sample, respectively.

After calculating the triplets for each quality attribute of the six milk‐barberry drinks, the triplets were incorporated into Equation (5) thus to determine the relative weighting of sensory scores pertaining to each sensory item:

(5) $$S_{r}O=S_{r}C\times {\text{QC}}_{\mathrm{rel}}+S_{r}T\times {\text{QT}}_{\mathrm{rel}}+S_{r}A\times {\text{QA}}_{\mathrm{rel}}+S_{r}M\times {\text{QM}}_{\mathrm{rel}}$$

where C is for color, T is for taste, A is for aroma, M is for mouth feel, the subscript “r” is for sample number, QC_rel_, QA_rel_, QT_rel_, and QM_rel_ represent the triplets associated with the relative weighting of quality characteristics of milk‐barberry drinks in general. For example, in the case of the color item, QCrel = SC/Qsum, where Qsum is the sum of the first digit of the triplets (Routray & Mishra, 2012).

#### 2.6.3 Assessment of membership function for standard fuzzy scale

Membership values of Membership function for each triangular distribution pattern of a 6‐point scale (illustrated and named F1, F2, F3, F4, F5, and F6) were defined by a set of 10 numbers as shown in Equation (6).

(6) $$\begin{array}{cc} F1 & =(1\;0.5\;0\;0\;0\;0\;0\;0\;0\;0)\\ F2 & =(0.5\;1\;1\;0.5\;0\;0\;0\;0\;0\;0)\\ F3 & =(0\;0\;0.5\;1\;1\;0.5\;0\;0\;0\;0)\\ F4 & =(0\;0\;0\;0\;0.5\;1\;1\;0.5\;0\;0)\\ F5 & =(0\;0\;0\;0\;0\;0\;0.5\;1\;1\;0.5)\\ F6 & =(0\;0\;0\;0\;0\;0\;0\;0\;0.5\;1) \end{array}$$

#### 2.6.4 Estimation of overall membership function of sensory scores on standard fuzzy logic scale

Overall membership function value of various samples was calculated, using one of the following equations:

(7) $$\begin{array}{cc} B_{x} & =\frac{x-(a-b)}{b},\;\mathrm{for}\;\left(a-b\right)<x<a\\ B_{x} & =\frac{(a+c)-x}{c},\;\mathrm{for}\;a<x<\left(a+c\right)\\ B_{x} & =1\;\text{for}\;x=a\\ B_{x} & =0\;\text{for}\;\text{all}\;\text{other}\;\text{values}\;\text{of}\;x \end{array},$$

where B_x_ is the value of membership function of sensory scores that are estimating at x = 0 to 100; then a, b and c are membership values of the sensory overall scores for the triplet of each sample. The x value can be included in the form of a set with ten numbers starting from 0 <  x < 10 to 90 <  x < 100 with intervals of 10, whereby the maximum values of B_x_ occurred in the mentioned range of x (Sinija & Mishra, 2011).

#### 2.6.5 Estimation of similarity values and the ranking of the milk‐barberry‐based drinks

After obtaining the B values for each of the samples on a standard fuzzy scale (Equation (7)), the similarity values of each individual sample was obtained by Equation (8):

(8) $$S_{m}\left(F,B\right)=\frac{F\times B^{\prime }}{\text{Max}(F\times F^{\prime }\text{and}B\times B^{\prime })},$$

where S_m_ represents the similarity value of a particular sample, the F' and B' represent the transpose of matrix F and B, respectively.

After determining the various similarity values, they were compared with each other to find out the maximum similarity value of each sample on six categories (viz. not at all necessary, extremely important and, etc.) of sensorial scales and, accordingly, all six milk‐barberry drinks were ranked. Matlab 2015a (The Mathworks Inc., Natick, MA) was used for the fuzzy logic analysis of the sensory data (Sinija & Mishra, 2011).

## 3 Results and Discussions

### 3.1 Chemical properties of barberry concentrate

Preliminary tests conducted on the BJC revealed its significant antioxidant activity. Different anthocyanins constitute the majority of pigments in the barberry fruit. In this study, the BJC's anthocyanin content was evaluated by the pH Differential Method on the basis of barberries' dominant anthocyanin. The total anthocyanins were 150.90 ± 0.02 (mg cyanidin‐3‐glucoside/100 ml). The amounts of anthocyanins were observed to increase after concentrating the juice solution. This is due to copigmentation, copolymerization, and acylation. Total phenolic contents, antioxidant activity and vitamin C were 282.25 ± 0.02 (mg GAE/100 ml), 85.90 ± 0.67 (%) and 69.55 ± 0.20 (mg/L), respectively.

Among natural antioxidants, phenolic compounds are a group of special interest because of their wide distribution in the plant kingdom. All the phenolic classes (including the simple phenolics, flavonoids, phenolic acids, and anthocyanins) have the structural requirements of free radical scavengers and have the potential to be used as food antioxidants (Sun, Chu, Wu, & Liu, 2002). Furthermore, vitamin C is a good contributor to the total antioxidant capacity of the food (Chaovanalikit & Wrolstad, 2004). The chemical analysis of BJC revealed the existence of considerable levels of antioxidant activity in this study, which is in agreement with a previous report by Hanachi et al. regarding relevant results on the antioxidant activity of *B. vulgaris* fruits (Hanachi, Kua, Asmah, Motalleb, & Fauziah, 2006).

### 3.2 Physiochemical properties of milk‐barberry drinks

#### 3.2.1 pH, dry solid and serum separation

From this study, the best formulation was optimized based on pH (optimized values of 3.5–4.2), dry solid (>%14) and serum separation (Table 2). Results showed that among 24 formulations, the S19 ‐ S24 were able to create gel strengthened networks, and the formulations coming S6, S9, S10, S11, and S12 exhibited nonappropriate properties due to precipitation (>%10) after 10 days. Other formulas including the S1, S2, S3, S7, S8, S13, S14, and S19 were not in the optimized pH range (3.5–4.2) at the beginning of production and thus were removed from the experiment. Therefore, just the S3, S4, S5, S16, S17, and S18 formulations were chosen among the 24 formulae for the application of sensory evaluations based on desirability values of pH, serum separation and viscosity.

**Table 2:** Physiochemical properties (in 3 replicates) of different prepared formulation in order to select the best formula of drink

Formulation code	pH	Total solid (%)	Serum separation	
			After homogenization	Before homogenization
S1	5.60 ± 0.00^a^	18.53 ± 0.01^s^	0.00 ± 0.00^k^	0.50 ± 0.06^k^
S2	4.63 ± 0.00^c^	19.22 ± 0.01^o^	6.00 ± 0.02^h^	7.00 ± 00.05^h^
S3	4.31 ± 0.00^d^	19.93 ± 0.02^l^	8.10 ± 0.31^f^	9.11 ± 0.01^fg^
S4	3.82 ± 0.00^e^	20.65 ± 0.02^i^	8.30 ± 0.35^f^	8.50 ± 0.10^f^
S5	3.65 ± 0.00^f^	21.36 ± 0.02^f^	7.00 ± 0.10^g^	10.00 ± 0.00^g^
S6	3.59 ± 0.00^fg^	22.14 ± 0.01^c^	11.00 ± 0.05^c^	12.00 ± 0.50^c^
S7	5.59 ± 0.01^a^	18.62 ± 0.01^r^	18.00 ± 0.10^a^	19.00 ± 0.00^a^
S8	4.61 ± 0.00^c^	19.29 ± 0.01^n^	7.50 ± 0.06^g^	8.50 ± 0.02^g^
S9	4.31 ± 0.00^d^	20.00 ± 0.02^k^	10.00 ± 0.10^d^	11.5 ± 0.50^de^
S10	3.81 ± 0.01^e^	20.71 ± 0.01^hi^	11.00 ± 0.07^c^	13.00 ± 0.00^c^
S11	3.60 ± 0.00^fg^	21.43 ± 0.01^e^	10.00 ± 0.03^d^	11.00 ± 0.07^d^
S12	3.51 ± 0.01^hi^	22.21 ± 0.01^b^	14.00 ± 0.25^b^	16.00 ± 0.00^b^
S13	5.57 ± 0.00^ab^	18.71 ± 0.02^q^	10.00 ± 0.11^d^	1.0000.01^d^
S14	4.60 ± 0.00^c^	19.35 ± 0.02^mn^	2.00 ± 0.06^j^	4.00 ± 0.04^j^
S15	4.30 ± 0.02^d^	20.07 ± 0.01^j^	4.00 ± 0.10^i^	6.10 ± 0.02^i^
S16	3.81 ± 0.00^e^	20.77 ± 0.03^h^	2.00 ± 0.15^j^	4.00 ± 0.05^j^
S17	3.58 ± 0.00^g^	21.51 ± 0.02^d^	4.10 ± 0.10^i^	6.00 ± 0.07^i^
S18	3.50 ± 0.00^hi^	22.29 ± 0.01^a^	9.00 ± 00.50^e^	9.70 ± 0.07^e^
S19	5.52 ± 0.01^b^	18.79 ± 0.01^p^	0.00 ± 0.00^k^	0.00 ± 0.00^k^
S20	4.59 ± 0.00^c^	19.41 ± 0.02^m^	0.00 ± 0.00^k^	0.00 ± 0.00^k^
S21	4.09 ± 0.10^d^	20.13 ± 0.01^j^	0.00 ± 0.00^k^	0.00 ± 0.00^k^
S22	3.79 ± 0.01^e^	20.85 ± 0.05^g^	0.00 ± 0.00^k^	0.00 ± 0.00^k^
S23	3.56 ± 0.00^gh^	21.57 ± 0.02^d^	0.00 ± 0.00^k^	0.00 ± 0.00^k^
S24	3.49 ± 0.01^i^	22.35 ± 0.02^a^	0.00 ± 0.00^k^	0.00 ± 0.06^k^

Different letters in each column indicate a significant difference (*p* < .05).

**Figure 2:**
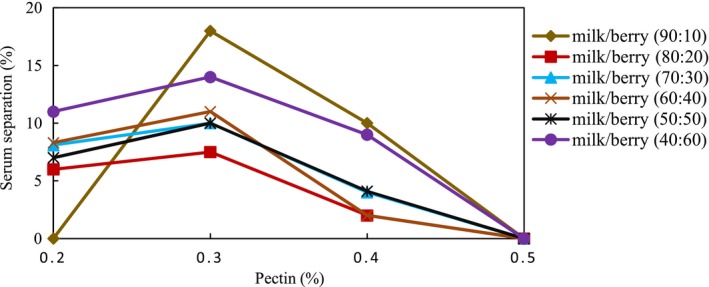
The stability of acidified milk drinks with various pectin concentrations

As it was mentioned previously, the interaction between biopolymers happened depending on different factors, among which pH was the most important. Different mechanisms including thermodynamic compatibility and thermodynamic incompatibility result in serum separation. For the sample containing the milk‐barberry with a ratio of (9:10), the most important destabilization mechanism is thermodynamic incompatibility due to having high pH values. For the other samples having lower values of pH, the electrostatic interaction occurs between milk protein and pectin; therefore, a higher mixing ratio (having a higher amount of pectin) results in more stability. A value of 0.2 pectin concentration for the samples containing approximately 10% BJC was not enough to impart phase separation via excluding volume effects. Increasing this value to 0.3% would result in competitive hydration between pectin and milk protein, thereby increasing the serum separation as a result of thermodynamic incompatibility. However, in the 0.4 pectin concentration, the viscosity of continuous phase increased and thereby reduced the serum separation. At the 0.5 pectin concentration, as the gel network was produced, the serum separation was prevented. Some trends were apparent in connection with the results of serum separation; serum separation in samples containing 0.2% pectin was less than samples which contained 0.3% pectin (Figure 2). As mentioned earlier, the protein–pectin interaction, and consequently the stability of AMDs depend on the concentration and type of pectin used, the concentration of casein and ionic strength, the pH, and the homogenization during processing (Glahn, 1982; Glahn & Rolin, 1994). Probably less than a 0.3% value of pectin concentration was insufficient for the electrostatic interaction to form the soluble complex with all casein micelles which were only partially covered, so that the protein can be linked by polymer bridges. However, the 0.5% concentration of HMP at low pH adsorbs onto casein protein surfaces as the result of electrostatic reaction and protein contact is prevented by steric hindrance; therefore, this forms a self‐supporting network which promotes the stability of the colloidal system (Tromp, de Kruif, van Eijk, & Rolin, 2004). Furthermore, the stabilization might be caused by a combination of depletion interactions among the complex of pectin/casein micelles and a pectin network (Tromp et al., 2004). Moreover, results revealed that homogenization reduced the serum separation samples throughout the whole studied period. This may be attributed to particle size reduction and the increase in pectin/casein bonding via homogenization. Pertinently, numerous studies have shown the intense influence of particles size on the stability of acidified milk (Sedlmeyer, Brack, Rademacher, & Kulozik, 2004).

#### 3.2.2 Viscosity

**Figure 3:**
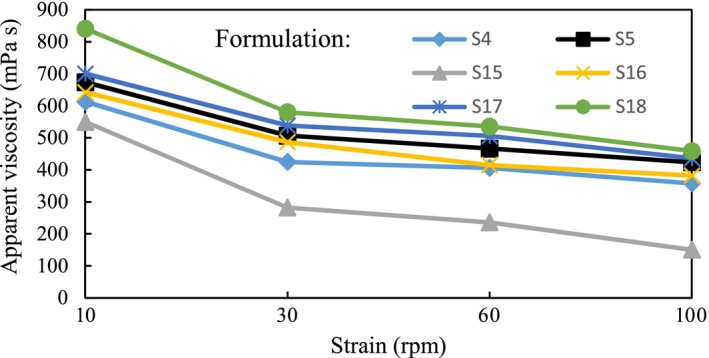
The variation of apparent viscosity of optimum formula as function of rotational speed; S4: milk‐barberry ratio (6:4) containing 0.2% pectin, S5: milk‐barberry ratio (5:5) containing 0.2% pectin, S15: milk‐barberry ratio (7:3) containing 0.4% pectin, S16: milk‐barberry ratio (6:4) containing 0.4% pectin, S17: milk‐barberry ratio (5:5) containing 0.4% pectin, S18: milk‐barberry ratio (4:6) containing 0.4% pectin. It is necessary to mention that in all formulations, fixed amount of sugar was considered (i.e., equal to 8 g)

Increasing the rotational rate from 10 rpm to 100 rpm led to the decrease in apparent viscosity of all samples (formulation S1 to S18) and thus resulted in a verified shear thinning behavior of samples. Furthermore, by increasing the pectin concentration and reducing the milk‐barberry ratio, the apparent viscosity increased. Figure 3 shows how apparent viscosity of selected formulations were optimized based on pH (optimized 3.5–4.2), dry solid (>%14) and serum separation (Table 2).

#### 3.2.3 Color analysis

Results of different variance analysis of the best formulations were optimized based on pH (optimized 3.5–4.2), dry solid (>%14) and serum separation with regard to the color index (L*, a*, b*) as shown in Table 3.

**Table 3:** Color measurement results of optimized formulations

Formulations^a^	Color measurement		
	*L* ^*^	*a* ^*^	*b* ^*^
Milk: Barberry ratio (6:4),pectin 0.2 g	33.00 ± 0.00^c,^ b	32.00 ± 0.05^c^	22.50 ± 0.40^c^
Milk: Barberry ratio (5:5),pectin 0.2 g	29.40 ± 0.04^d^	39.00 ± 0.08^b^	28.00 ± 0.00^b^
Milk: Barberry ratio (7:3),pectin 0.4 g	53.25 ± 0.04^a^	26.27 ± 0.03^d^	18.00 ± 0.25^d^
Milk: Barberry ratio (6:4),pectin 0.4 g	39.60 ± 0.04^b^	32.09 ± 0.09^c^	22.10 ± 0.10^c^
Milk: Barberry ratio (5:5),pectin 0.4 g	38.70 ± 0.00^b^	39.16 ± 0.16^b^	28.00 ± 0.14^b^
Milk: Barberry ratio (4:6),pectin 0.4 g	32.50 ± 0.30^c^	40.15 ± 0.00^a^	30.00 ± 0.30^a^

a It is necessary to mention that in all formulations, fixed amount of sugar was considered (i.e., equal to 8 g).

b Different letters in each column indicate a significant difference (*p* < .05).

The highest and the lowest magnitudes of lightness (L*) were observed, respectively, in the M: B ratio (7: 3) containing 0.4% pectin and in the M: B ratio (5:5) containing 0.2% pectin. In the case of yellowness index (b*) and redness index (a*), the lowest and highest values pertained, respectively, to the M: B ratio (7: 3) containing 0.4% pectin and the M: B ratio (4:6) containing 0.4% pectin. Results indicated a positive correlation between the barberry concentrate and a* and b*, due to the existence of pigments and components like carotenoids and anthocyanins which make the color of barberry. On the contrary, the correlation between L* and the barberry concentrate was negative, indicating the decrease in milk with high lightness.

#### 3.2.4 Sensory analysis of drinks using fuzzy logic

The sensory response obtained from the panel group of panelists for the screened milk‐barberry samples (samples S3, S4, S15, S16, S17, and S18) are presented in Table 4. It can be observed that the panelists ranked the S4 for color, S16 for taste, S15, and S16 for mouth feel as Very Good/Excellent by greater proportions. Sensory score triplets of each sample were calculated through Equation (4) which is given in Table 4.

**Table 4:** Panelists Preference for specific quality characteristics of *milk‐barberry* samples and triplets related with sensory scores

Sensory attributes of samples	Sensory scale factors and corresponding numerical values					Sensory scores triplet
	Poor (0 0 25)^a^	Fair (25 25 25)	Good (50 25 25)	Very good (75 25 25)	Excellent (100 25 0)	
Color/appearance						
S3	0	9	9	2	0	(41.25 25 25)
S4	0	0	3	12	5	(77.5 25 18.75)
S15	0	11	9	0	0	(36.25 25 25)
S16	0	0	4	10	6	(77.5 25 17.5)
S17	0	2	11	5	2	(58.75 25 22.5)
S18	0	4	11	4	1	(52.5 25 23.75)
Taste						
S3	1	8	6	2	1	(37.5 21.25 21.25)
S4	0	1	9	7	3	(65 25 21.25)
S15	2	7	7	4	0	(41.25 22.5 25)
S16	0	1	8	9	3	(70 26.25 22.5)
S17	0	6	10	2	2	(50 25 22.5)
S18	3	7	5	2	3	(43.75 21.25 21.25)
Aroma/Smell						
S3	0	5	7	6	2	(56.25 25 22.5)
S4	0	3	9	4	4	(61.25 25 20)
S15	0	5	8	5	2	(55 25 22.5)
S16	0	2	9	6	3	(62.5 25 21.25)
S17	0	3	8	6	3	(61.25 25 21.25)
S18	0	2	9	5	4	(63.75 25 20)
Mouth feel						
S3	0	4	10	6	0	(52.5 25 25)
S4	0	5	8	6	1	(53.75 25 23.75)
S15	0	2	6	8	4	(67.5 25 20)
S16	0	2	6	9	3	(66.5 25 21.25)
S17	0	6	10	3	1	(48.75 25 23.75)
S18	0	7	11	2	0	(43.75 25 25)
Overall						
S3	(46.861 37.609 29.737)					
S4	(64.126 43.659 29.855)					
S15	(50.213 38.841 30.045)					
S16	(68.954 45.344 30.195)					
S17	(54.511 40.851 30.077)					
S18	38.786 29.561)					

a Triplets related with 5‐point sensory scale.

The sensory responses provided by the panelists, the sensory score triplet, and the triplet of the corresponding relative weighting of each quality attribute are displayed in Table 5. Results show that none of the sensory quality attributes are given the “not at all important” scores. According to the graphical representation of membership function of a triplet (a, b, c), the value of membership function is close to 1 when the value “a” is large enough and/or the value “c” is small (Figure 1). Therefore, the taste is the strongest quality of milk–barberry samples, while color is the weakest. The important quality attributes of *milk‐barberry* samples in general were rated according to the following order of importance: taste > mouth feel > aroma > color. This result supports findings by several previous investigations, which indicated that taste is the most important quality attribute (Fatma, Sharma, Singh, Jha, & Kumar, 2016; Meena et al., 2015). Also, Routray and Mishra (2012) defined quality attributes in the dahi drinks according to the following order of importance: Taste > Flavor > Homogeneity > Color (Routray & Mishra, 2012). Therefore, these results can play a significant role in the optimization of ingredients that are intended to be incorporated into the milk‐barberry samples.

**Table 5:** Sum of individual preference to the importance of quality attributes of samples in general

	NI	SI	I	HI	EI	Sensory score triplet	Triplets for relative weightage
Color/appearance	0	1	3	6	10	(81.25 25 12.5)	(0.2355 0.0725 0.0362)
Taste	0	0	0	8	12	(90 25 10)	(0.2609 0.0725 0.0290)
Aroma	0	0	2	8	10	(85 25 12.5)	(0.2464 0.0725 0.0362)
Mouth feel	0	0	0	9	11	(88.75 25 11.25)	(0.2572 0.0725 0.0326)

NI*‐ not at all important, SI*‐ somewhat important, I*‐ important, HI*‐ highly important, EI*‐ extremely important.

For determination of overall sensorial scores of all samples, Equation (5) was used and the results are given in Table 4. Overall membership function values of various samples (Table 6) and values of membership function of standard fuzzy scale (nominated as F1, F2, etc.) were used for the determination of similarity values of milk‐barberry samples (Equation (8)). Similarity values for six milk‐barberry samples in different scale factors are shown in Table 7. Bold‐faced texts and highlights show the highest similarity value for each drink.

**Table 6:** Overall membership function value of various samples

Overall membership function			Value							
B1 (3)	0.01990	0.28580	0.55200	0.81760	1.00000	0.89440	0.55818	0.22190	0.0000	0.00000
B2 (4)	0.00000	0.00000	0.21840	0.44741	0.67640	0.90550	1.00000	0.80324	0.46830	0.13340
B3 (15)	0.00000	0.22213	0.47960	0.73710	0.99450	1.00000	0.67420	0.34140	0.00860	0.00000
B4 (16)	0.00000	0.00000	0.14090	0.36145	0.58200	0.80250	1.00000	0.96530	0.63420	0.30300
B5 (17)	0.00000	0.15520	0.40000	0.64480	0.88960	1.00000	0.81750	0.48500	0.15250	0.00000
B6 (18)	0.00000	0.20750	0.46530	0.72310	0.98100	1.00000	0.68670	0.34840	0.01010	0.00000

The maximum similarity value obtained under the category “good” were observed in (S17: 0.755047) and (S16: 0.723721). For sample S4, the highest similarity value was under the category “Very good” and for S3, S15 and S18 the highest similarity value became part of the category “Satisfactory”. After the comparison of the highest similarity values of samples, the ranking was ordered out as follows: Sample S4 (very good) > Sample S17 (good) > Sample S16 (good) > Sample S3 (satisfactory) > Sample S15 (satisfactory) > Sample S18 (satisfactory). Thus, it was clear that milk‐barberry samples with 40% and 50% barberry extract were the most acceptable sample of drink in the set of the samples. Overall, the drinks prepared with the addition of 0.4% pectin were better than those with 2% pectin sample. The addition of pectin could enhance the textural properties and mouth feel of drinks, and the addition of barberry extract improves the flavor. Drinks with 60% concentration of barberry extract were the least acceptable among the other six samples. The addition of flavor to milk has been found to increase the popularity of dairy products in food markets (Routray & Mishra, 2012). However, the results revealed that as the concentration of barberry extract increased beyond 50%, a sour aftertaste ensued after drinking, which was not acceptable by the majority of panelists. The classification of all samples into satisfactory, good and very good also implies that all samples have an acceptable quality for consumers. Therefore, the fuzzy analysis of sensory attributes demonstrated a good ability in ranking the milk‐barberry samples (Table 7). This technique has been successfully used for mango drinks (Jaya & Das, 2003), sausage (Lee & Kwon, 2007), Dahi‐Based Drinks (Routray & Mishra, 2012), bread (Singh, Mishra, & Mishra, 2012) instant green tea powder (Sinija & Mishra, 2011) kheer mohan (Meena et al., 2015), Beetroot Candy (Fatma et al., 2016) and gluten‐free pasta (Sakre, Das, & Srivastav, 2015).

**Table 7:** Similarity values for milk‐barberry samples

Sensory scale	1 (S3)	2 (S4)	3 (S15)	4 (S16)	5 (S17)	6 (S18)
F1 (NS)	0.050684	0.000000	0.032811	0.000000	0.023200	0.030857
F2 (fair)	0.390858	0.129771	0.316583	0.090500	0.264538	0.309766
F3 (satisfactory)	**0.790112**	0.494715	**0.730712**	0.397695	0.264538	**0.730078**
F4 (good)	0.641480	0.539342	0.692285	**0.723721**	**0.755047**	0.704614
F5 (very good)	0.155784	**0.725778**	0.203074	0.632378	0.456964	0.210306
F6 (excellent)	0.000000	0.107751	0.001182	0.174255	0.022898	0.001498
Rank	VI	I	IV	III	II	V

Bold‐faced texts show the highest similarity value for each drink; NS: not satisfactory.

## 4 Conclusion

Effects of pectin were investigated on the stability of the milk‐barberry drink in the presence and absence of homogenization. Results show that high methoxyl pectin at a concentration around 0.4% (w/w) was the best concentration for the stabilization of milk‐barberry drinks in the pH range of 3.5–4.2 and rheological properties showed that the stabilized samples behaved as shear thinning liquids, with viscosity increasing parallel to the increase in pectin and barberry juice content. Sensory evaluation was performed for six screened milk‐barberry samples by a panel of 20 panelists, and the data were analyzed, using the fuzzy logic method. It was found that the important quality attributes of milk‐barberry samples in general were rated according to the following order: taste > mouth feel > aroma > color. Overall acceptance values of each sample were determined by the maximum similarity value. Sample S4 (milk: barberry ratio (6:4) containing 0.2% pectin) and sample S3 (Milk‐barberry ratio (7: 3) containing 0.2% pectin) have the highest and the lowest acceptance, respectively. Therefore, Fuzzy analysis of sensory attributes has a good potential for ranking milk‐barberry samples.

## Conflict of Interest

None declared.
